# In-Hospital Palliative Care: Should We Need to Reconsider What Role Hospitals Should Have in Patients with End-Stage Disease or Advanced Cancer?

**DOI:** 10.3390/jcm7020018

**Published:** 2018-01-30

**Authors:** Paolo Cotogni, Andrea Saini, Anna De Luca

**Affiliations:** Pain Management and Palliative Care, Department of Anesthesia and Intensive Care, Molinette Hospital and University of Turin, C.so Bramante 88/90, 10126 Turin, Italy; a.saini@libero.it (A.S.); adeluca@cittadellasalute.to.it (A.D.L.)

**Keywords:** palliative care team, seriously ill patients, end-of-life, quality of life, symptom relief, acute palliative care unit, cost savings

## Abstract

Traditionally, palliative care (PC) systems focused on the needs of advanced cancer patients, but most patients needing PC have end-stage organ diseases. Similarly, PC models focus on the needs of patients in hospices or at home; however, in most cases PC is provided in acute hospitals. Indeed, the symptom burden that these patients experience in the last year of life frequently forces them to seek care in emergency departments. The majority of them are admitted to the hospital and many die. This issue poses important concerns. Despite the efforts of attending healthcare professionals, in-hospital patients do not receive optimal care near the end-of-life. Also, evidence is emerging that delay in identifying patients needing PC have a detrimental impact on their quality of life (QoL). Therefore, there is an urgent need to identify, early and properly, these patients among those hospitalized. Several trials reported the efficacy of PC in improving the QoL in these patients. Each hospital should ensure that a multidisciplinary PC team is available to support attending physicians to achieve the best QoL for both PC patients and their families. This review discusses the role and the impact of in-hospital PC in patients with end-stage disease or advanced cancer.

## 1. Palliative Care: For Who?

The World Health Organization defines palliative care as: “an approach that improves the quality of life of patients and their families facing the problem associated with life-threatening illness, through the prevention and relief of suffering by means of early identification and impeccable assessment and treatment of pain and other problems, physical, psychosocial and spiritual” [1].

Aging of the population as well as advances in medicine and public health are currently increasing the prevalence of people affected by progressive, chronic, life-limiting illnesses who would benefit of a palliative care (PC) approach. This phenomenon represents a great challenge for the healthcare systems especially in terms of patients’ assistance and healthcare costs [2].

Traditionally, PC services have been focused on the needs of cancer patients with advanced or metastatic disease. As a matter of fact, in-hospital PC programs have also begun to pay attention to on the needs of patients affected by non-oncological diseases, such as end-stage organ failure (heart failure, chronic obstructive pulmonary disease (COPD), liver cirrhosis, and severe renal dysfunction), as well as neurological illnesses (amyotrophic lateral sclerosis, Parkinson’s disease, multiple sclerosis, Alzheimer’s, and other dementias) [3,4]. It is also important to underline that patients with cancer or heart, chronic obstructive pulmonary, and renal diseases at an advanced stage have been demonstrated to share a similar spectrum of symptoms, and have a common pathway during the last period of their illness trajectories [5]. Therefore nowadays, the area of action of PC is the relief of symptoms as well as psychological, social, and spiritual issues of patients with chronic life-limiting diseases, regardless of the diagnosis [3].

## 2. Why Do Patients with Chronic Serious Illness Visit the Emergency Department near the End of Life?

During the last 6–12 months of life, most patients with end-stage disease or advanced cancer experience an increase in symptom burden which forces them to seek care in acute hospitals. These patients are usually admitted to the emergency department (ED) in order to administer them urgent care for the relief of uncontrolled symptoms [6]. Frequently, the visit may be precipitated by the distress of family members near the end of life (EoL) [7].

A recent study, focused on PC symptom management, has identified pain, dyspnea, nausea and vomiting, anorexia, anxiety, and constipation as the most frequent symptoms in seriously ill patients who are admitted to the ED, regardless of the specific disease. In addition, dehydration and acute urinary retention are other common symptoms of these patients when they come to an ED [8].

Nearly 50% of patients experience uncontrolled pain at the EoL [5]. Worsening dyspnea is also frequent in patients in need of PC. Diseases commonly causing severe dyspnea are acute exacerbations of COPD, lung cancer, and congestive heart failure. Frequent causes of increased shortness of breath are pneumonia, pulmonary embolism, pleural effusion, pericardial tamponade, pneumothorax, and superior vena cava syndrome. Nausea and vomiting are common gastrointestinal symptoms in these patients due to several causes: medications (opioids, chemotherapy agents), bowel obstruction, impaired gastric emptying, constipation, and increased intracranial pressure. Frequently, reduced oral food intake or severe weight loss due to anorexia (appetite loss), dysphagia, early satiety, stomatitis, and taste changes are common reasons for visits to the ED. Anxiety, confusion or delirium in seriously ill patients represent a quite frequent cause of access to the ED, as its management in home setting can be very difficult. Constipation is found in up to 90% of PC patients, regardless of the underlying illness. Constipation is typically caused by opioid intake, but also by bowel obstruction in patients bearing peritoneal carcinomatosis or advanced intra-abdominal tumors.

Sometimes, patients with end-stage disease or advanced cancer need (in the ED) of procedures such as intravenous fluids infusion for dehydration, placement of a urinary catheter for acute urinary retention, paracentesis for symptomatic intractable ascites or thoracentesis for symptomatic severe pleural effusion.

## 3. How to Identify Patients in Need of Palliative Care?

Unfortunately, in the medical literature there is no agreement about the definition of the ‘palliative care patient’ [9]. Evidence is emerging that delay or lack in identifying patients in need of PC have a potential harmful impact on their care [10]. In fact, a delayed recognition of those patients is associated with inadequate continuity of care, insufficient support, and increased healthcare costs [11]. Therefore, there is an urgent need to early and properly identify among the hospitalized seriously ill patients and those in need of PC.

The ED should be considered as a critical point of PC access for patients with end-stage disease or advanced cancer [12,13]. In our study, we found that more than one-third of people affected by these chronic diseases awaiting to be hospitalized after an ED visit should be identified as in need of PC consultation [14]. For cancer patients, we took into account the indicators elaborated by the National Comprehensive Cancer Network (NCCN) [15] to identify patients in need of PC, while we followed the criteria proposed by the Italian Society of Anesthesia Analgesia Resuscitation and Intensive Care (SIAARTI) [16] for patients with end-stage diseases. This study demonstrated the feasibility of applying NCCN/SIAARTI screening tools to identify patients in the ED for referral to a PC team.

The identification of PC needs in the ED, at the beginning of the hospital course, may provide a greater benefit to patients, families, and hospitals because it lets the PC consultation happen earlier rather than later in the patient’s hospital course, as it usually occurs. Wu et al. showed that PC consultation in the ED was associated with a significantly shorter length of stay for patients admitted to the hospital [17]. Grudzen et al. demonstrated that an ED-initiated PC consultation improved quality of life (QoL) without shortening survival in advanced cancer patients [18].

## 4. What Role Is There for In-Hospital Palliative Care?

Historically, PC models focused on the needs of patients in hospice or at home. However, many patients with end-stage disease or advanced cancer experience repeated unplanned hospital admissions, particularly in the last year of life [19]. In our study, we observed that about 75% of the patients affected by these diseases in need of PC, awaiting hospitalization after admission to the ED, had more than one in-hospital admission during the last year [14]. Moreover, in high-income countries the majority of patients with end-stage disease or advanced cancer die in hospitals [20] and this issue poses important concerns. Therefore, we felt the need to provide a review about the hospital based PC. We searched MEDLINE (1995–2017) and our research included the following subject heading terms: cancer, serious illness, unmet needs, palliative care delivery, quality of EoL care, and healthcare costs. To be included in this review, articles had to refer to in-hospital patients.

In 1995, the first large controlled trial investigating the EoL decision-making in seriously ill hospitalized patients was published [21]. The aim of this study was to understand prognoses and preferences for outcomes and risks of treatments (SUPPORT trial) in 9105 adults hospitalized patients with life-threatening diagnoses. The five analyzed outcomes were: (1) pain; (2) days in an intensive care unit (ICU), comatose or receiving mechanical ventilation before death; (3) hospital resource use; (4) incidence and timing of written do-not-resuscitate (DNR) orders; and (5) patient-physician agreement on cardiopulmonary resuscitation preferences. The authors concluded that the care of seriously ill or dying hospitalized patients was not optimal.

The care of patients with chronic life-limiting illnesses has been recognized in the recent years as suboptimal in hospital settings [22,23,24,25]. Particularly, patients and their families often complain of inadequate pain relief and other symptoms as well as futile diagnostic imaging and unwanted life-sustaining treatments at the EoL [26,27,28].

Another unmet need is the communication between the attending physicians and the patients/relatives. This communication is often inadequate in explaining complex medical information including prognosis and treatment options, resulting in misunderstandings mainly at the EoL [29,30]. Moreover, the referral to the hospice is implemented by physicians far too late in the course of the illness, even though it was observed that family members reported lower satisfaction with delayed hospice services [31,32].

Muir and Arnold reported that dying patients in acute care hospitals were often in physical pain, without attention to their emotional and spiritual suffering [33]. The authors emphasized that hospital physicians should become the primary providers of PC for these hospitalized patients with end-stage diseases.

The weak point of this model is that the majority of hospital healthcare professionals do not currently receive proper education on communication, especially concerning EoL care in dying patients. In fact, medical and nursing schools worldwide have traditionally had a limited emphasis on EoL care [34]. However, PC and EoL issues are not an easy conversation between the attending physician and the patient or caregivers. Consequently, many of them report that they have encountered important ethical dilemmas in managing refractory symptoms, discontinuing life-sustaining therapies in dying patients [35], and, especially, considering palliative sedation [36]. In this respect, a closer collaboration with university to enhance the healthcares’ knowledge about management of refractory symptoms (particularly, pain) and EoL care would be useful.

Evidence from clinical studies suggest that patients with end-stage diseases are often treated during hospitalization with aggressive medical intervention, including prolonged ICU stay, rather than with PC [22,28]. Often, the care these patients receive during their ICU stay is more aimed to prolong their lives, rather than to the palliation of their symptoms [37]. Similarly, aggressive EoL care in the last weeks of life of advanced cancer patients, such as chemotherapy administration or ICU admission are commonly deemed markers of poor quality care [38].

It is of concern that caregivers of patients receiving aggressive care are at higher risk of experiencing a major depressive disorder, and in general a worse QoL, likely because they are unprepared for the death of their relatives [39]. Several studies pointed out that one barrier to providing quality care at the EoL was the lack of communication between the patient/family and the medical team [40]. It has been shown that most patients would like to have a discussion with their physicians about EoL care, but this often remained an unmet issue [41,42].

In order to better manage patients with end-stage disease or advanced cancer in recent years hospital-based PC programs have been developed [43,44]. Specifically, the aim of these programs is to improve symptom management, help patients and families to better understand prognosis and treatment options, clarify goals of care, and assist in planning of care as the disease progresses.

Several studies indicated that hospital-based PC services increased satisfaction of patients and families [45], improved QoL [46], reduced ICU length of stay [47], and decreased costs [48,49]. In a very recent review, the authors have underlined the advantages of a PC-oriented approach in improving patient’s outcomes, including symptom burden, QoL, and EoL outcomes, all achieved with lower associated costs [50].

## 5. How Do In-Hospital Palliative Care Services Work?

Within hospitals, the primary model of PC delivery is the multidisciplinary consultation team, that should be composed of physicians, nurses, and psychologists trained in PC, with the support of specialty physicians (geriatrician, internist, physiatrist, neurologist, psychiatrist) and other healthcare professionals (dietician, physical therapist, respiratory therapist) as well as pharmacists, social workers, case managers, spiritual counselors, and volunteers [51]. The PC team must have a tight bidirectional communication with home PC services, hospices, and primary care physicians.

Indeed, in the United States a PC team is present in almost 90% of hospitals with 300 beds or more and two thirds of hospitals with 50 beds or more [51]. During the last decade, also in Europe, PC programs in hospitals have significantly increased, although much remains to be done in this area.

The PC team provides consultations to inpatients, working with attending healthcare professionals to ensure patient-focused and family-centered care. The most important purpose of the team is to achieve the best possible QoL for both patients and their families/caregivers by assessing and managing refractory symptoms [52]. Also, the team helps both patients’ and family’s understanding of the underlying disease process and promotes decision-making based on informed choices. Actually, the team should be involved in those cases where management of symptoms requires more than the basic knowledge or communication becomes difficult due to arising conflicts.

Both randomized controlled trials and observational studies compared the outcomes of patients with chronic life-limiting diseases who were referred to hospital-based PC teams to those of patients who received usual care. According to these studies the patients referred to PC reported reduced symptom burden, improved QoL, and decreased spiritual distress [53,54,55,56].

In recent years, a new approach in managing patients with end-stage diseases in the hospital context has been represented by the acute palliative care unit (APCU) [57]. The focus of the APCU is rapid symptom control and intensive psychosocial care, with a shorter length of stay and a lower death rate (20–50%) compared to in hospice ones [58]. Therefore, the APCU differs from a hospice which offers more extensive long-term PC or exclusively near-death care.

The APCU provides intensive PC for in-hospital patients and families, with the aim of enhancing QoL, facilitating transition to EoL care, and assisting with hospital discharge. The discharge from the APCU represents an important and complex process, as it must provide a location (e.g., home, hospice, or long-term care facility) where the patient finds appropriate care until death, avoiding unplanned hospital admissions for lack of symptom management skills [10]. The choice of the discharge location depends on availability of caregivers and, mainly, on the patient’s preferences, performance status, and life expectancy as well as other logistic and financial factors [59].

In short, the APCU may be the hospital place to provide patients with end-stage disease or advanced cancer with a personalized medicine tailored to their individual needs.

## 6. What Is the Impact of In-Hospital Palliative Care Services on Cost Savings?

The increasing development of medical technology gives in-hospital physicians the opportunity to easily opt for life-prolonging therapies or devices in patients with end-stage disease or advanced cancer; e.g., new combination of adjuvant chemotherapy with targeted drugs, left ventricular-assisted devices, mechanical ventilation, haemodialysis, and artificial nutrition. Consequently, these patients frequently undergo various imaging techniques, interventional procedures, and therapies, even near the end of their lives. Such physicians’ behavior does not translate in a better management of the PC patient nor in a better patient’s QoL, and it rather often causes more discomfort to the patients and a source of unnecessary costs for the healthcare system.

Several studies suggest that PC consultation is associated with cost saving in hospitalized patients, as it allows to identify those patients who would benefit from a palliative approach rather than an aggressive one. Since 2008, Morrison et al. stated that hospital PC team consultations were associated with significant hospital cost savings [60]. Starks et al. reported that PC team interventions resulted in cost savings for short and medium length of stay, but not for stays longer than 30 days. Therefore, the authors concluded that cost savings can be achieved by an earlier call on the PC team [61].

In a recent review Dalal and Bruera have reported that the magnitude of hospital cost savings with PC involvement ranged from 9% to 32% and these savings were higher when a PC team was involved earlier (within two days from the patient’s admission) [50]. Two recent studies [62,63] have pointed out that cancer patients’ care was characterized by high-cost and low-value interventions, high hospital readmission rates, prolonged length of stay, hospital deaths, and frequent use of chemotherapy and other disease-centered interventions near the EoL. These data are indicative of poor care coordination and inadequate EoL planning, and such a management translates into cost escalation, while patients continue to receive non-beneficial and burdensome health interventions at the EoL. These studies demonstrated that referral to the PC team improved various healthcare utilizations and quality measures, including 30-day readmission rates, hospice use, and avoiding chemotherapy following discharge.

## 7. Conclusions

The attitudes and knowledge of healthcare professionals towards PC may exert influence on their communication with patients and caregivers and, mainly, on patients’ quality-of-care near the EoL. Therefore, the best management of PC for hospitalized patients with end-stage disease or advanced cancer should be based on a well-defined cooperation between PC teams and treating physicians. Actually, multidisciplinary PC teams aim to address the full spectrum of patients’ health, spiritual, and psychosocial needs, and match treatment choices with patients’ values and will.

In conclusion, we sincerely believe that to improve the care of these inpatients with relevant symptom burdens, particularly after an unplanned hospital admission or when the patient is dying, each hospital should ensure that professionals expert in PC are available to support treating physicians.

## References

[1] World Health Organization Definition of Palliative Care. http://www.who.int/mediacentre/factsheets/fs402/en/.

[2] Gòmez-Batiste X., Martinez-Munozm M., Blaym C., Espinosa J., Contel J.C., Ledesma A. (2012). Identifying needs and improving palliative care of chronically ill patients: A community-oriented, population-based, public-health approach. Curr. Opin. Support. Palliat. Care.

[3] Higginson I.J., Addington-Hall J.M. (1999). Palliative care needs to be provided on basis of needs rather than diagnosis. BMJ.

[4] Connor S.R., Sepulveda Bermedo M.C. (2014). Global Atlas of Palliative CARE at the End of Life.

[5] Solano J.P., Gomes B., Higginson I.J. (2006). A comparison of symptom prevalence in far advanced cancer, AIDS, heart disease, chronic obstructive pulmonary disease and renal disease. J. Pain Symptom Manag..

[6] Barbera L., Taylor C., Dudgeon D. (2010). Why patients with cancer visit the emergency department near the end of life?. CMAI.

[7] Smith A.K., Fisher J., Schonberg M.A., Pallin D.J., Block S.D., Forrow L., Phillips R.S., McCarthy E.P. (2008). Am I doing the right thing? Provider perspectives on improving palliative care in the emergency department. Ann. Emerg. Med..

[8] Siegel M., Biegelow S. (2018). Palliative care symptom management in the emergency department: The ABC’s of symptom management for the emergency physician. J. Emerg. Med..

[9] Van Mechelen W., Aertgeerts B., De Ceulaer K., Thoonsen B., Vermandere M., Warmenhoven F., Van Rijswijk E., De Lepeleire J. (2013). Defining the palliative care patient: A systematic review. Palliat. Med..

[10] Gardiner C., Ingleton C., Gott M., Ryan T. (2011). Exploring the transition from curative care to palliative care: A systematic review of the literature. BMJ Support. Palliat. Care.

[11] Gott M., Ingleton C., Bennett M.I., Gardiner C. (2011). Transitions to palliative care in acute hospitals in England: Qualitative study. BMJ.

[12] Bruera E. (2016). Emergency department point of palliative care access for patients with advanced cancer. JAMA Oncol..

[13] George N.R., Kryworuchko J., Hunold K.M., Ouchi K., Berman A., Wright R., Grudzen C.R., Kovalerchik O., LeFebvre E.M., Lindor R.A. (2016). Shared decision making to support the provision of palliative and end-of-life care in the emergency department: A consensus statement and research agenda. Acad. Emerg. Med..

[14] Cotogni P., De Luca A., Evangelista A., Filippini C., Gili R., Scarmozzino A., Ciccone G., Brazzi L. (2017). A simplified screening tool to identify seriously ill patients in the emergency department for referral to a palliative care team. Minerva Anestesiol..

[15] NCCN (2017). Clinical Practice Guidelines in Oncology (NCCN Guidelines^®^): Palliative Care. https://www.nccn.org/professionals/physician_gls/pdf/palliative.pdf.

[16] SIAARTI (2013). Italian Society of Anaesthesia Analgesia Resuscitation and Intensive Care. Grandi Insufficienze D’organo “End Stage”: Cure Intensive o Cure Palliative? “Documento Condiviso” per una Pianificazione Delle Scelte di Cura. http://www.siaarti.it/News/grandi-insufficienze-organo-end-stage-cure-intensive-o-cure-palliative.aspx.

[17] Wu F.M., Newman J.M., Lasher A., Brody A.A. (2013). Effects of initiating palliative care consultations in the emergency department on inpatient length of stay. J. Palliat. Med..

[18] Grudzen C.R., Richardson L.D., Johnson P.N., Hu M., Wang B., Ortiz J.M., Kistler E.A., Chen A., Morrison R.S. (2016). Emergency department-initiated palliative care in advanced cancer: A randomized clinical trial. JAMA Oncol..

[19] Cotogni P., De Luca A., Saini A., Brazzi L. (2017). Unplanned hospital admissions of palliative care patients: A great challenge for internal and emergency medicine physicians. Intern. Emerg. Med..

[20] Cohen J., Bilsen J., Addington-Hall J., Lofmark R., Miccinesi G., Kaasa S., Deliens L. (2008). Population-based study of dying in hospital in six European countries. Palliat. Med..

[21] JAMA (1995). A controlled trial to improve care for seriously ill hospitalized patients. The study to understand prognoses and preferences for outcomes and risks of treatments (SUPPORT). The SUPPORT Principal Investigators. JAMA.

[22] Ahronheim J.C., Morrison R.S., Baskin S.A., Morris J., Meier D.E. (1996). Treatment of the dying in the acute care hospital. Advanced dementia and metastatic cancer. Arch. Intern. Med..

[23] Baker R., Wu A.W., Teno J., Kreling B., Damiano A.M., Rubin H.R., Roach M.J., Wenger N.S., Phillips R.S., Desbiens N.A. (2000). Family satisfaction with end-of-life care in seriously ill hospitalized adults. J. Am. Geriatr. Soc..

[24] Mularski R.A., Heine C.E., Osborne M.L., Ganzini L., Curtis J.R. (2005). Quality of dying in the ICU: Ratings by family members. Chest.

[25] Teno J.M., Clarridge B.R., Casey V., Welch L.C., Wetle T., Shield R., Mor V. (2004). Family perspectives on end-of-life care at the last place of care. JAMA.

[26] Claessens M.T., Lynn J., Zhenshao Z., Desbiens N.A., Phillips R.S., Wu A.W., Harrell F.E., Connors A.F. (2000). Dying with lung cancer or chronic obstructive pulmonary disease. Insights from SUPPORT. J. Am. Geriatr. Soc..

[27] Lynn J., Teno J.M., Phillips R.S., Wu A.W., Desbiens N., Harrold J., Claessens M.T., Wenger N., Kreling B., Connors A.F. (1997). Perceptions by family members of the dying experience of older and seriously ill patients. SUPPORT Investigators. Study to Understand Prognoses and Preferences for Outcomes and Risks of Treatments. Ann. Intern. Med..

[28] Teno J.M., Fisher E., Hamel M.B., Wu A.W., Murphy D.J., Wenger N.S., Lynn J., Harrell F.E. (2000). Decision making and outcomes of prolonged ICU stay in seriously ill patients. J. Am. Geriatr. Soc..

[29] Weiner J.S., Cole S.A. (2004). Three principles to improve clinician communication for advance care planning: Overcoming emotional, cognitive, and skill barriers. J. Palliat. Med..

[30] Tulsky J.A. (2005). Beyond advance directives: Importance of communication skills at the end of life. JAMA.

[31] Miller S.C., Kinzbrunner B., Pettit P., Williams J.R. (2003). How does the timing of hospice referral influence hospice care in the last days of life?. J. Am. Geriatr. Soc..

[32] Shocklett E.R., Teno J.M., Miller S.C., Stuart B. (2005). Late referral to hospice and bereaved family member perception of quality of end-of-life care. J. Pain Symptom Manag..

[33] Muir J.C., Arnold R.M. (2001). Palliative care and the hospitalist: An opportunity for cross-fertilization. Am. J. Med..

[34] Block S.D. (2002). Medical education in end-of-life care: The status of reform. J. Palliat. Med..

[35] Kinzbrunner B.M. (1995). Ethical dilemmas in hospice and palliative care. Support. Care Cancer.

[36] Cotogni P., Brazzi L. (2017). Palliative sedation: A feasible option to improve end-of-life care in seriously ill dying patients. Minerva Anestesiol..

[37] Au D.H., Udris E.M., Fihn S.D., McDonell M.B., Curtis J.R. (2006). Differences in health care utilization at the end of life among patients with chronic obstructive pulmonary disease and patients with lung cancer. Arch. Intern. Med..

[38] Rocque G.B., Barnett A.E., Illig L., Eickhoff J.C., Bailey H.H., Vampbell T.C., Stewart J.A., Cleary J.F. (2013). Inpatient hospitalization of oncology patients: Are we missing an opportunity for end-of-life care?. J. Oncol. Pract..

[39] Wright A.A., Zhang B., Ray A., Mack J.W., Trice E., Balboni T., Mitchell S.L., Jackson V.A., Block S.D., Maciejewski P.K. (2008). Associations between end-of-life discussions, patient mental health, medical care near death, and caregiver bereavement adjustment. JAMA.

[40] Mack J.W., Smith T.J. (2012). Reasons why physicians do not have discussions about poor prognosis, why it matters, and what can be improved. J. Clin. Oncol..

[41] Hagerty R.G., Butow P.N., Ellis P.M., Lobb E.A., Pendlebury S.C., Leighl N., MacLeod C., Tattersall M.H. (2005). Communicating with realism and hope: Incurable cancer patients’ views on the disclosure of prognosis. J. Clin. Oncol..

[42] Steinhauser K.E., Christakis N.A., Clipp E.C., McNeilly M., Grambow S., Parker J., Tulsky J.A. (2001). Preparing for the end of life: Preferences of patients, families, physicians, and other care providers. J. Pain Symptom Manag..

[43] Manfredi P.L., Morrison R.S., Morris J., Goldhirsch S.L., Carter J.M., Meier D.E. (2000). Palliative care consultations: How do they impact the care of hospitalized patients?. J. Pain Symptom Manag..

[44] Pan C.X., Morrison R.S., Meier D.E., Natale D.K., Goldhirsch S.L., Kralovec P., Cassel C.K. (2001). How prevalent are hospital-based palliative care programs? Status report and future directions. J. Palliat. Med..

[45] Dy S.M., Shugarman L.R., Lorenz K.A., Mularski R.A., Lynn J. (2008). RAND-Southern California Evidence-Based Practice Center. A systematic review of satisfaction with care at the end of life. J. Am. Geriatr. Soc..

[46] Morrison R.S. (2005). Health care system factors affecting end-of-life care. J. Palliat. Med..

[47] Back A.L., Li Y.F., Sales A.E. (2005). Impact of palliative care case management on resource use by patients dying of cancer at a Veterans Affairs medical center. J. Palliat. Med..

[48] Brumley R., Enguidanos S., Jamison P., Seitz R., Morgenstern N., Saito S., McIlwane J., Hillary K., Gonzalez J. (2007). Increased satisfaction with care and lower costs: Results of a randomized trial of in-home palliative care. J. Am. Geriatr. Soc..

[49] Gade G., Venohr I., Conner D., McGrady K., Beane J., Richardson R.H., Williams M.P., Liberson M., Blum M., Della Penna R. (2008). Impact of an inpatient palliative care team: A randomized control trial. J. Palliat. Med..

[50] Dalal S., Bruera E. (2017). End-of-life care matters: Palliative cancer care results in better care and lower costs. Oncologist.

[51] Kelly A.S., Morrison R.S. (2015). Palliative care for the seriously ill. N. Engl. J. Med..

[52] Wilkinson E.K., Salisbury C., Bosanquet N., Franks P.J., Kite S., Lorentzon M., Naysmith A. (1999). Patient and carer preference for, and satisfaction with, specialist models of palliative care: A systematic literature review. Palliat. Med..

[53] Higginson I.J., Finlay I., Goodwin D.M., Cook A.M., Hood K., Edwards A.G., Douglas H.R., Norman C.E. (2002). Do hospital-based palliative teams improve care for patients or families at the end of life?. J. Pain Symptom Manag..

[54] Bakitas M., Lyons K.D., Hegel M.T., Balan S., Brokaw F.C., Seville J., Hull J.G., Li Z., Tosteson T.D., Byock I.R. (2009). Effects of a palliative care intervention on clinical outcomes in patients with advanced cancer: The Project ENABLE II randomized controlled trial. JAMA.

[55] Temel J.S., Greer J.A., Muzikansky A., Gallagher E.R., Admane S., Jackson V.A., Dahlin C.M., Blinderman C.D., Jacobsen J., Pirl W.F. (2010). Early palliative care for patients with metastatic non-small-cell lung cancer. N. Engl. J. Med..

[56] Balboni T.A., Vanderwerker L.C., Block S.D., Paulk M.E., Lathan C.S., Peteet J.R., Prigerson H.G. (2007). Religiousness and spiritual support among advanced cancer patients and associations with end-of-life treatment preferences and quality of life. J. Clin. Oncol..

[57] Eti S., O’Mahony S., McHugh M., Guilbe R., Blank A., Selwyn P. (2014). Outcomes of the acute palliative care unit in an academic medical centre. Am. J. Hosp. Palliat. Med..

[58] Shin S.H., Hui D., Chisholm G.B., Kwon J.H., San Miguel M.T., Allo J.A., Yennurajalingam S., Frisbee-Hume S.E., Bruera E. (2014). Characteristics and outcomes of patients admitted to an acute palliative care unit from the emergency centre. J. Pain Symptom Manag..

[59] Hui D., Elsayem A., Palla S., De La Cruz M., Li Z., Yennurajalingam S., Bruera E. (2010). Discharge outcomes and survival of patients with advanced cancer admitted to an acute palliative care unit at a comprehensive cancer centre. J. Palliat. Med..

[60] Morrison R.S., Penrod J.D., Cassel J.B., Caust-Ellenbogen M., Litke A., Spragens L., Meier D.E. (2008). Palliative Care Leadership Centers’ Outcomes Group. Cost savings associated with US hospital palliative care consultation programs. Arch. Intern. Med..

[61] Starks H., Wang S., Farber S., Owens D.A., Curtis J.R. (2013). Cost savings vary by length of stay for inpatients receiving palliative care consultation services. J. Palliat. Med..

[62] Adelson K., Paris J., Horton J.R., Hernandez-Tellez L., Ricks D., Morrison R.S., Smith C.B. (2017). Standardized criteria for palliative care consultation on a solid tumor oncology service reduces downstream health care use. J. Oncol. Pract..

[63] Triplett D.P., LeBrett W.G., Bryant A.K., Bruggeman A.R., Matsuno R.K., Hwang L., Boero I.J., Roeland E.J., Yeung H.N., Murphy J.D. (2017). Effect of palliative care on aggressiveness of end-of-life care among patients with advanced cancer. J. Oncol. Pract..

